# Transarterial chemoembolization (TACE) plus tyrosine kinase inhibitors versus TACE in patients with hepatocellular carcinoma: a systematic review and meta-analysis

**DOI:** 10.1186/s12957-023-02961-7

**Published:** 2023-03-31

**Authors:** Ruihua Duan, Fen Gong, Yan Wang, Caixia Huang, Jiaming Wu, Leihao Hu, Min Liu, Shijun Qiu, Liming Lu, Yisheng Lin

**Affiliations:** 1 First Clinical Medical College, Guangzhou University of Chinese Medicine, Guangzhou, China; 2 Department of Radiology, The First Affiliated Hospital of Guangzhou University of Chinese Medicine, Guangzhou, China; 3 Medical Examination Center, Huizhou Central People’s Hospital, Huizhou, Guangdong China; 4 Zhongshan Affiliated Hospital, Guangzhou University of Chinese Medicine, Zhongshan, China; 5 Department of Interventional Radiology, The First Affiliated Hospital of Guangzhou University of Traditional Chinese Medicine, 16 Jichang Road, Guangzhou, China; 6 Medical College of Acupuncture-Moxibustion and Rehabilitation, Guangzhou University of Chinese Medicine, Guangzhou, China; 7 Dongguan Institute of Guangzhou University of Chinese Medicine, Dongguan, 523808 China; 8 Department of Interventional Radiology, Huizhou Municipal Central Hospital, Huizhou Guangdong, China

**Keywords:** Tyrosine kinase inhibitors, Hepatocellular carcinoma, TACE, Systematic review, Meta-analysis

## Abstract

**Purpose:**

Transarterial chemoembolization (TACE) with tyrosine kinase inhibitors (TKIs) has been increasingly used to treat unresectable hepatocellular carcinoma (uHCC). However, the superiority of combination therapy to TACE monotherapy remains controversial. Therefore, here we performed a meta-analysis to evaluate the efficacy and safety of TACE plus TKIs in patients with uHCC.

**Methods:**

We searched four databases for eligible studies. The primary outcome was time to progression (TTP), while the secondary outcomes were overall survival (OS), tumor response rates, and adverse events (AEs). Pooled hazard ratios (HRs) with 95% confidence intervals (95% CIs) were collected for TTP and OS, and the data were analyzed using random-effects meta-analysis models in STATA software. OR and 95% CIs were used to estimate dichotomous variables (complete remission[CR], partial remission[PR], stable disease[SD], progressive disease[PD], objective response rate[ORR], disease control rate[DCR], and AEs) using RStudio’s random-effects model. Quality assessments were performed using the Newcastle–Ottawa scale (NOS) for observational studies and the Cochrane risk of bias tool for randomized controlled trials (RCTs).

**Results:**

The meta-analysis included 30 studies (9 RCTs, 21 observational studies) with 8246 patients. We judged the risk of bias as low in 44.4% (4/9) of the RCTs and high in 55.6% (5/9) of the RCTs. All observational studies were considered of high quality, with a NOS score of at least 6. Compared with TACE alone or TACE plus placebo, TACE combined with TKIs was superior in prolonging TTP (combined HR 0.72, 95% CI 0.65–0.80), OS (combined HR 0.57, 95% CI 0.49–0.67), and objective response rate (OR 2.13, 95% CI 1.23–3.67) in patients with uHCC. However, TACE plus TKIs caused a higher incidence of AEs, especially hand-foot skin reactions (OR 87.17%, 95%CI 42.88–177.23), diarrhea (OR 18.13%, 95%CI 9.32–35.27), and hypertension (OR 12.24%, 95%CI 5.89–25.42).

**Conclusions:**

Our meta-analysis found that TACE plus TKIs may be beneficial for patients with uHCC in terms of TTP, OS, and tumor response rates. However, combination therapy is also associated with a significantly increased risk of adverse reactions. Therefore, we must evaluate the clinical benefits and risks of combination therapy. Further well-designed RCTs are needed to confirm our findings.

**Trial registration:**

PROSPERO registration number: CRD42022298003.

**Supplementary Information:**

The online version contains supplementary material available at 10.1186/s12957-023-02961-7.

## Introduction

Hepatocellular carcinoma (HCC) is the sixth most common cancer worldwide and the third leading cause of cancer-related death. Primary liver cancers include HCC (75–85% of cases) and intrahepatic cholangiocarcinoma (10–15%). The incidence of HCC is increasing annually. More than 700,000 people are diagnosed with HCC each year, and more than half of these cases occur in developing countries, especially Asian countries [1]. The main risk factors for HCC are chronic infection with hepatitis B or C virus, exposure to aflatoxin-contaminated food, alcohol consumption, overweight, type 2 diabetes, and smoking [2].

Due to the high incidence of advanced HCC, palliative care aimed at prolonging life after diagnosis is an important component of its management [3]. Many therapies have been used to treat advanced HCC, including transarterial chemoembolization (TACE), radiation, immunotherapy, systemic chemotherapy, portal vein stenting, percutaneous ethanol injection, and conservative therapy [4–10].

TACE is a local treatment strategy for the palliative treatment or management of most unresectable HCC (uHCC) patients that aims to prevent and alleviate patient suffering and improve their quality of life. A reported 55% of patients achieved partial remission (PR) after TACE treatment, significantly delaying tumor progression and macrovascular invasion [11]. However, TACE induces hepatocyte hypoxia by blocking the blood vessels and upregulating vascular endothelial growth factor (VEGF) in normal and tumor cells. High expression of hypoxia-inducible factor-1α can alter VEGF stability and enhance its mRNA expression. Hypoxia can also induce high VEGF receptors expression in endothelial cells. Binding of VEGF to VEGF receptors leads to angiogenesis, promotes vascular remodeling, and can lead to residual tumor cell growth, which plays an important role in HCC local recurrence or metastasis [12]. Therefore, inhibitors targeting the VEGF signaling pathway have become the main approach for tumor therapy. Tyrosine kinase inhibitors (TKIs) inhibit the activation of downstream signaling pathways (RAS MAPK, PI3K AKT, and JAK STAT) and prevent the proliferation and migration of HCC cells by binding to the corresponding kinases phosphorylated by their substrate tyrosine residues, invasion, and angiogenesis [13]. The survival benefit of a VEGF TKI in a variety of solid tumors was observed in a series of randomized clinical trials (RCTs). Therefore, the combination of antiangiogenic drugs and TACE therapy may improve the therapeutic effect [14–16].

With the development and application of TKIs in the treatment of cancer, TACE combined with TKIs for the treatment of liver cancer has become a hot topic in clinical research. Preliminary clinical research results are represented by TACE plus sorafenib, and many studies have shown that the combined treatment effect is better [17]. TACE plus TKI combination therapy provides additional options, such as combining apatinib, lenvatinib, brivanib, orantinib, and sorafenib. However, some clinical trial results showed that orantinib combined with TACE does not improve overall survival in patients with uHCC [18]; the clinical trial results showed that TACE plus lenvatinib significantly improved clinical outcomes versus TACE monotherapy [19]. Therefore, it remains controversial whether TACE plus TKIs is superior to TACE monotherapy.

This systematic review and meta-analysis aimed to analyze the safety and efficacy of TACE plus TKIs for the treatment of uHCC.

## Materials and methods

### Search strategy

This meta-analysis was conducted according to the PRISMA guidelines [20]. We searched the PubMed, Embase, Cochrane Library, and Web of Science databases from inception to March 15, 2022. We established search strategies that combined database-specific subject headings (such as MeSH terms) and free text terms (such as hepatocellular carcinoma/liver cancer/hepatoma, TACE/transcatheter arterial chemoembolization/transarterial chemoembolization, orantinib or TSU-68/sorafenib/lenvatinib/apatinib/brivanib, randomized clinical trials/clinical trials) to identify potentially eligible studies. Studies not published in English were also excluded. Letters, commentaries, editorials, and case reports were also excluded. Potential studies were reviewed by two independent reviewers. If there was any uncertainty about eligibility, a third reviewer was consulted.

### Study inclusion and exclusion criteria

The inclusion criteria were as follows: (1) study design: RCTs, retrospective or prospective cohort studies, and case control studies; (2) study population: patients with uHCC; (3) intervention: TACE plus sorafenib/lenvatinib/apatinib/brivanib/orantinib versus TACE plus placebo or TACE alone (including conventional TACE and TACE with drug-eluting beads); and (4) the study was limited to English language articles and required adult patient information including overall survival (OS) and time to progression (TTP) (HR and corresponding 95% confidence interval [CI]), tumor response rates, and adverse events (AEs).

Studies were excluded if they met the following criteria: (1) comments, editorials, systematic reviews, meta-analyses, and studies unrelated to our topics were excluded from the final analysis, as were those unrelated to our topic or lacking useful information; (2) the same study was published by the same authors or based on the same database; (3) cases treated with TACE combined with other anti-tumor drugs were excluded; and (4) cases treated with TACE combined with TKIs and immunotherapy were excluded.

### Data extraction

The following information was extracted from studies that met the following inclusion criteria: study characteristics (author name, year of publication, study design, sample size), population characteristics (mean age, sex, country, Barcelona Clinical Liver Cancer [BCLC] stage, Child–Pugh score, Eastern Oncology Collaboration Group [ECOG], hepatitis), intervention characteristics (median drug treatment period, dose, tumor response (objective response rate [ORR], disease control rate [DCR], complete remission [CR], partial response[PR], stable disease [SD], progressive disease [PD], and outcomes [OS, TTP, and safety]). Two independent reviewers extracted the data, and a third reviewer resolved any discrepancies.

### Quality assessment

**Fig. 1:**
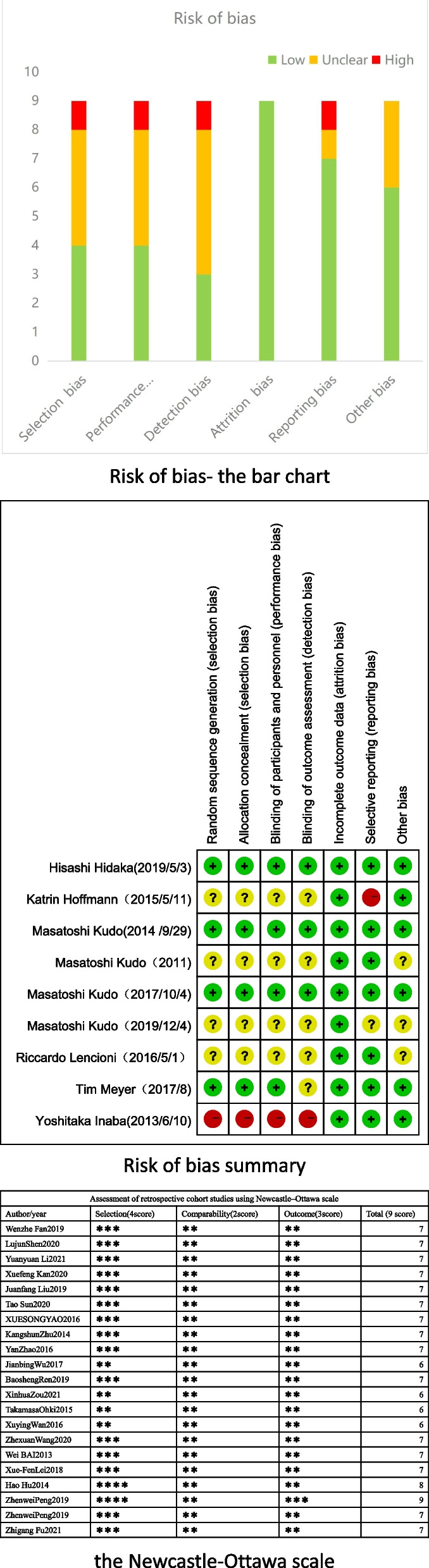
Risk-of-bias graph for randomized controlled trials and observational studies

Two reviewers independently assessed the quality of non-randomized controlled studies (RCTs) using the Newcastle–Ottawa scale (NOS) [21], which contains three domains: (1) selection, (2) comparability, and (3) outcome. The maximum NOS score was 9 points, and a score ≥ 6 indicated a high-quality study (Additional file 1: Table 1). The Cochrane risk of bias tool 5.1.0 was applied to examine RCTs [22] using a grading scheme for each of its six main aspects: (1) selection bias, (2) performance bias, (3) detection bias, (4) attrition bias, (5) reporting bias, and (6) other bias. These six were further graded, and each part was evaluated as one of the following levels: “low risk of bias, unclear risk of bias,” and “high risk of bias.” A study was assessed as high quality if four or more parts were assessed as having a low risk of bias (Fig. 1).

### Statistical analysis

We assessed the overall efficacy of TACE plus TKIs in the treatment of patients with HCC based on data from the included studies. For the time-to-event variables including overall survival (OS), TTP, HRs with 95% CI were directly extracted, and HR values were combined. Pooled HR estimates were calculated using a fixed effects model. However, when heterogeneity was relatively large, the random-effects model was used to summarize the pooled data. Odds ratios (OR) were used to estimate dichotomous variables (CR, PR, SD, PD, ORR, DCR, and AEs), both with corresponding 95% CI.

A test for heterogeneity, defined as the variation between individual trials for a given treatment rather than that expected from chance, was used to assess whether the magnitude of a given treatment effect varied between the trials. The *I*^*2*^ statistic describes the percentage of total variation across studies owing to heterogeneity rather than chance. Studies with an* I*^*2*^ value of < 25%, 50%, 75%, and 100% were considered to have no, low, moderate, and high heterogeneity, respectively.

Publication bias was evaluated using Begg’s and Egger’s tests [19, 20]. Values of *p* < 0.05 were considered statistically significant. Funnel plots were used to assess publication bias. Statistical analyses were performed using STATA version 14.0 (Stata Corporation, College Station, TX, USA), Review Manager (Revman, version 5.3.0, The Cochrane Collaboration, 2012), and R (version 4.1.2) within the RStudio (2021.09.1) platform.

### Evidence certainty

The Grading of Recommendations Assessment, Development, and Evaluation (GRADE) tool was used to assess the overall quality and strength of available evidence [23]. Details of the GRADE evidence profile are shown in Additional file 2: Table 2.

## Results

### Study selection and quality assessment

**Fig. 2:**
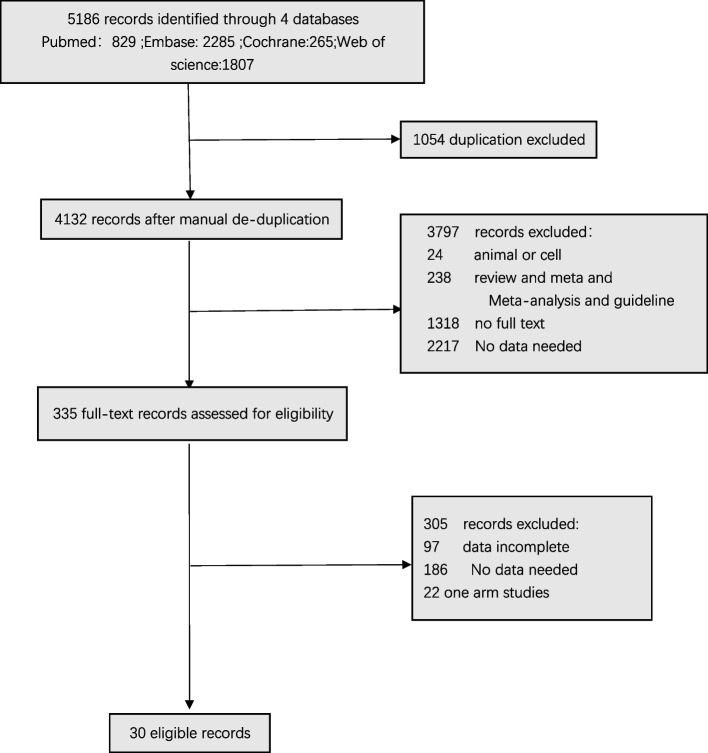
Study selection

The literature search yielded a total of 5186 studies for screening. The preliminary review excluded 1054 articles, while the title and abstract screening excluded 3797 studies. Thus, 335 articles were subjected to full-text review. Our analysis ultimately included 30 studies: nine (30%) RCTs and 21 (70%) observational studies. A screening flowchart for this study is shown in Fig. 2 [18, 19, 24–51].

### Study characteristics

The baseline characteristics of the 30 studies included in the meta-analysis are presented in Table 1. These 30 studies were published between 2011 and 2021, including 20 in China [19, 26–32, 35, 38, 39, 41, 43–49, 51], 1 in South Korea [40], 7 in Japan [18, 24, 25, 33, 37, 40, 50], 1 in Germany [42], 1 in the USA [36], and 1 in the UK [34], The studies included a total of 8246 patients with uHCC, including 4423 in the combination group and 3823 in the TACE alone or TACE plus placebo, with a total of 48 to 1719 patients each study. The mean age of the studies was 40–73 years, and the majority of patients were men (Table 1). The majority of patients selected had (BCLC) stage A or B, an ECOG physical fitness status (PS) score of 0 or 1, and Child–Pugh grade A or B. The TTP for TACE plus TKIs and TACE alone groups was 71–801 days and 51–492 days, respectively. The OS of the TACE plus TKIs and TACE alone groups was 210–1086 days and 147–990 days, respectively.

**Table 1:** Characteristics of included studies in the meta-analysis and systematic review

Author	Country	Treatment	Mean age (years)	Male/female	Study design	Number of cases	Median treatment period (days)	BCLC stage (%)	Child–Pugh (%)	ECOG (%)	Viral hepatitis (%) (HBV, HCV)
Hisashi Hidaka 2019	Japan	TACE + orantinib VS TACE + placebo	71 VS 71	Male: 178 VS 176 Female: 41 VS 37	RCT	219 VS 213	298	0: 2.7% VS 4.2% A: 29.7% VS 25.4% B: 55.3% VS 55.9% C: 12.3% VS 14.1%	A: 100% VS 100%	0: 94.5% VS 91.5% 1: 5.5% VS 8.5%	HbsAg positive:17.8% VS 14.1% HCVAb positive:59.8% VS 57.3%
Masatoshi Kudo 2017	Japan	TACE + orantinib VS TACE + placebo	66·2 VS 65·4	Male: 363 VS 364 Female:81 VS 80	RCT	445 VS 444	327	0: 2% VS 3% A: 33% VS 27% B: 47% *VS* 52% C: 17% VS 16%	A: 100% VS 100%	0: 90% VS 91% 1: 10% VS 9%	HbsAg positive: 38% VS 45% HbsAb positive: 24% VS 20% HbcAb positive: 70% VS 68% HCV positive:43% VS 37%
Yoshitaka Inaba 2013	Japan	TACE + orantinib VS TACE-alone	NA	Male: 39 VS 43 Female: 11 VS 8	RCT	50 VS 51	122	0: 6.0% VS 17.6% A: 36.0% VS 25.5% B: 48.0% VS 52.9% C: 10.0% VS 10.0%	A: 80.0% VS 88.2% B: 18.0% VS 11.8% unknown: 2.0% VS 0.0%	0: 90.0% VS 96.1% 1: 10.0% VS 3.9%	HbsAg positive: 4.0% VS 7.8% HbcAb positive:80.0% VS 70.6%
Tao Sun 2020	China	TACE + apatinib VS TACE-alone	55.56 ± 5.2 VS 58.65 ± 6.6	Male: 24 VS 21 Female:3 VS 10	Retrospective controlled study	27 VS 31	NA	C: 100% VS 100%	A:77.8% VS 74.2% B: 22.2% VS 25.8%	1: 77.8% VS 80.6% 2: 22.2% VS 19.4%	B: 92.6% VS 90.3%
Wenzhe Fan 2019	China	TACE + apatinib VS TACE-alone	49 VS 50	Male: 68 VS 71 Female:17 VS 32	Retrospective controlled study	85 VS 103	NA	B or C: 100% VS 100%	A: 85.9% VS 84.5% B: 14.1% VS 15.5%	0: 78.8% VS 87.4% 1–2: 21.2% VS 12.6%	B: 81.9% VS 75.7%
Xuefeng Kan 2020	China	TACE + apatinib VS TACE-alone	52.7 ± 9.7 VS 53.1 ± 10.1	Male: 77 VS 78 Female:13 VS 12	Retrospective controlled study	90 VS 90	NA	B: 88.9% VS 87.8%	A: 87.8% VS 85.6% B: 12.2% VS 14.4%	1: 82.2% VS 83.3% 2: 17.8% VS 16.7%	B: 88.9% VS 87.8%
Juanfang Liu 2019	China	TACE + apatinib VS TACE-alone	53.3 ± 9.4 VS 56.5 ± 9.7	Male: 29 VS 39 Female:5 VS 9	Retrospective controlled study	34 VS 48	NA	B:52.9% VS 58.3% C:47.1% VS 41.7%	A: 58.8% VS 60.4% B: 41.2% VS 39.6%	0–1: 47.1% VS 45.8% 2: 52.9% VS 54.2%	B: 64.7% VS 77.1% C:14.7% VS 10.4%
Yuanyuan Li 2021	China	TACE‑apatinib VS TACE‑125I	56.62 ± 10.1 VS 51.63 ± 9.9	Male: 19 VS 25 Female:2 VS 2	Retrospective controlled study	21 VS 27	NA	B or C: 100% VS 100%	A: 81.0% VS 66.7% B: 19.0% VS 33.3%	0:71.4% VS 74.1% 1:29.0% VS 25.9%	NA
Zhiyu Qiu 2019	China	TACE + apatinib VS TACE-alone	NA	Male: 41 VS 73 Female:1 VS 10	A Propensity Score Matching Analysis	42 VS 83	NA	B:21.4% VS 34.9% C:78.6% VS 65.1%	A:85.7% VS 90.4% B:14.3% VS 9.6%	NA	B: 92.9% VS 88.0%
Lujun Shen 2020	China	TACE + apatinib VS TACE-alone	NA	Male: 38 VS 74 Female:2 VS 6	Retrospective controlled study	40 VS 80	111	NA	A: 82.5% VS 80.0% B: 17.5% VS 20.0%	NA	B: 90.0% VS 93.8%
Masatoshi Kudo 2014	Japan	TACE + Brivanib VS TACE + placebo	57 VS 59	Male: 206 VS 216, female: 43 VS 37	RCT	249 VS 253	NA	A: 26% VS 23% B: 52% VS 59% C: 22% VS 17%	A: 96% VS 91% B: 4% VS 8% C: < 1% VS 1%	0: 80% VS 84% 1: 20% VS 16%	B: 63% VS 66% C: 20% VS 17%
Zhigang Fu 2021	China	TACE + lenvatinib VS TACE-alone	60 VS 60	Male: 50 VS 55, Female: 10 VS 5	Retrospective controlled study	60 VS 60	246.9	A:3.3% VS 5.0% B:55.0% VS 43.3% C:41.7% VS 51.7%	A:93.3% VS 95.0% B:6.7% VS 5.0%	NA	B:80.0% VS 80.0% C:3.3% VS 3.3%
Tim Meyer 2017	UK	DEB-TACE + sorafenib VS DEB-TACE + placebo	65 VS 68	Male: 139 VS 138, female: 18 VS 18	RCT	157 VS 156	120	NA	A: 100% VS 100%, (5) 68% vs 73% (6) 25% VS 22% (7) 3% VS 1% unknown: 4% VS 3%	0: 62%: 62% 1: 37%: 37% unknown: 1%: 1%	B: 5% VS 6%, C: 12% VS 6% B + C: 2% VS 2%
Xuesong Yao 2016	China	TACE + sorafenib VS TACE-alone	56.5 VS 55.9	Male: 44 VS 87, female: 6VS 13	Prospective nonrandomized controlled study	50 VS 100	NA	B: 42% VS 40%, C: 58% VS 60%	A: 84% VS 86%, B: 16% VS 14%	0: 42% VS 34% 1: 58% VS 66%	B: 84% VS 83% C: 4% VS 4% B + C: 4% VS 3%
Riccardo Lencioni 2016	USA	DEB-TACE + sorafenib VS DEB-TACE + placebo	64.5 VS 63.0	Male: 135 VS 126, female: 19 VS 27	RCT	154 VS 153	147	B: 100% VS 100%	A: (5) 63.6% VS 68.6%(6) 35.7% VS 30.7%(7) 0.6% VS 0, unknown: 0: 0.7%	0: 100% VS 100%	B:35.7% VS 32.7% C: 25.3% VS 26.8% B + C: 1.3% VS 0
Masatoshi Kudo 2019	Japan	TACE + sorafenib VS TACE-alone	72.0 VS 73.0	Male: 63 VS 55, female: 17 VS 21	RCT	80 VS 76	270.9	A:33.8% VS 43.4% B:55.0% VS 44.7% C:11.3% VS 11.8%	A: 98.8% VS 93.5% B: 1.3% VS 5.6%	0: 88.8% VS 88.2%, 1: 11.3% VS 11.8%	B: 12.5% VS 2.6% C: 47.5% VS 69.7%
Zhexuan Wang 2020	China	TACE + sorafenib VS TACE-alone	53.7 ± 12.0 VS 56.7 ± 12.1	Male: 267 VS 1183, female: 46 VS 223	Retrospective controlled study	1,406 VS 313	309	A:11.5% VS 13.7% B:53.3% VS 53.8% C:35.1% VS 32.6%	A: 95.6% VS 93.8% B: 4.5% VS 6.2%	0: 64.9% VS 67.4%, 1: 35.1% VS 32.6%	B: 83.1% VS 83.0% C: 5.1% VS 2.6%
Kangshun Zhu 2014	China	TACE + sorafenib VS TACE-alone	48.4 ± 8.1 VS 51.9 ± 12.2	Male: 39 VS 38, female: 7 VS 7	Retrospective controlled study	46 VS 45	330	NA	A: 84.7% VS 86.7% B: 15.2% VS 13.3%	0: 47.8% VS 44.4%, 1–2: 52.1% VS 55.6%	B: 82.3% VS 88.9% C: 10.9% VS 2.2%
Masatoshi Kudo 2011	Japan and Korean	TACE + sorafenib VS TACE + placebo	69 VS 70	Male: 174VS 168, female: 55 VS 61	RCT	229 VS 229	513	NA	NA	0: 87.8% VS 87.8% 1: 12.2% VS 12.2%	B: 20.5% VS 22.7% C: 60.7% VS 64.6%
Yan Zhao 2016	China	TACE + sorafenib VS TACE-alone	53 VS 54	Male: 159 VS 159, female: 24 VS 24	Multicenter retrospective controlled study	183 VS 183	489	NA	A: 97.3% VS 3.8% B: 97.3% VS 2.7%	0: 85.8% VS 14.2%, 1: 88.5% VS 11.5%	B/C: 88.0% VS 88.0%
Katrin Hoffmann 2015	Germany	TACE + sorafenib VS TACE + placebo	58.5 VS 58.0	45\5	RCT	24 VS 26	125	NA	A: 58.3% VS 83.3% B: 37.5% VS 23.1% C: 4.2% vs 0%	NA	B: 12.5% VS 11.5% C: 45.8% VS 26.9%
Jianbing Wu 2017	China	TACE + sorafenib VS TACE-alone	NA	Male: 25 VS 28, female: 2 VS 3	Retrospective controlled study	30 VS 31	NA	C: 100% VS 100%	A: 93.3% VS 80.6% B: 6.6% VS 6.5%	0: 80% VS 77.4%, 1: 20% VS 22.6%	B/C: 90% VS 96.8%
Hao Hu 2014	china	TACE + sorafenib VS TACE-alone	61 ± 11 VS 60 ± 11	Male: 69 VS 140, female: 13 VS 24	retrospective cohort study	82 VS 164	NA	NA	A: 70.7% VS 62.8% B: 29.3% VS 37.2%	NA	B: 82.9% VS 84.8% C: 7.3% VS 6.1%
Wei Bai 2013	China	TACE + sorafenib vs TACE-alone	54 ± 13 VS 52 ± 12	Male: 73 VS 146 female: 9 VS 18	Prospective nonrandomized controlled study	82 VS 222	NA	B: 23.2% VS 27.4% C: 76.8% VS 72.6%	A:76.8% VS 70.1% B:23.2% VS 29.9%	0: 36.6% VS 29.3% 1: 46.4% VS 61.6% 2: 14.6% VS 9.1% 3: 1.2% VS 0% 4: 1.2% VS 0%	B: 87.8% VS 89.6% C: 4.9% VS 4.3%
Zhenwei Peng 2019	China	TACE + sorafenib VS TACE-alone	55 ± 7.6 VS 56 ± 8.3	Male: 107 VS 110 female: 21VS 22	Retrospective cohort study	128 VS 132	NA	A: 80.4% VS 72.0%, B: 19.5% VS 28.0%	NA	NA	B: 82.0% VS 85.6% C: 4.7% VS 5.3%
Baosheng Ren 2019	China	TACE + sorafenib VS TACE-alone	NA	Male: 48 VS 102 female: 13 VS 20	Retrospective controlled study	61 VS 122	351	B: 49.2% VS 59.0%, C: 50.8% VS 41.0%	A: 90.1% VS 91.0%, B: 9.8% VS 9.0%	0: 59.0% VS 56.6%, 1–2: 41.0% VS 43.4%	B: 82.0% VS 76.2% C: 8.2% VS 7.3%
Xinhua Zou 2021	China	TACE + sorafenib VS TACE-alone	58.31 ± 7.83 VS 58.53 ± 8.11	Male: 32 VS 31 female: 10 VS 12	Retrospective controlled study	42 VS 43	NA	B: 54,8% VS 58.1%, C: 45.2% VS 41.9%	A: 69.0% VS 67.4%, B: 26.2% VS 30.2%, C: 4.8% VS 2.3%	0: 21.4% VS 23.3% 1: 69.0% VS 69.8, 2: 9.5% VS 7.0%	B: 54.8% VS 58.1% C: 45.2% VS 41.9%
Xue-Fen Lei 2018	China	TACE + sorafenib vs TACE-alone	52 ± 5 VS 51 ± 6	Male: 24 VS 18 female: 14 VS 11	Retrospective controlled study	38 VS 29	NA	B: 100% VS 100%	A:65.8% VS 65.5% B:34.2% VS 34.5%	0: 100% VS 100%	NA
Takamasa Ohki 2015	Japan	TACE + sorafenib vs TACE-alone	70.0 VS 72.9	Male: 20 VS 54 female: 4 VS 17	Retrospective controlled study	24 VS 71	412	NA	A:70.8% VS 29.2% B:56.3% VS 43.7%	NA	C: 75.0% VS 67.6%
Xuying Wan 2016	China	TACE + sorafenib vs TACE-alone	NA	Male: 218 VS 218 female: 27 VS 27	Retrospective controlled study	245 VS 245	324 ± 315.3	NA	A:86.6% VS 93.7% B:13.4% VS 6.3%	0/1: 90.6% VS 82.7% 2: 9.4% VS 17.3%	NA
Author	Alcohol hepatitis(%)	Viral hepatitis + alcohol hepatitis	Dose(mg)	ORR	DCR	CR	PR	SD	PD	TTP (days)	OS (days)
Hisashi Hidaka 2019	NA	NA	200, twice daily	NA	NA	NA	NA	NA	NA	141 VS 93, HR 0.76 (0.619, 0.940)	975 VS 990, HR 0.981 (0.717, 1.343)
Masatoshi Kudo 2017	NA	NA	200, twice daily	NA	NA	NA	NA	NA	NA	87 VS 75, HR 0.858 (0.744, 0.990)	933 VS 969, HR 1.09 ( 0.878, 1.352)
Yoshitaka Inaba 2013	NA	NA	200, twice daily	NA	NA	NA	NA	NA	NA	157 VS 122, HR 0.699 (0.450, 1.088)	780: unknown, HR 1.06 (0.578, 1.492)
Tao Sun 2020	NA	NA	500, twice daily	mRECIST: 37.0% VS 16.1%	62.9% VS 29.0%	0% VS 0%	37.0% VS 16.1%	25.9% VS 12.9%	37.0% VS 71.0%	270 VS 150, HR 0.56 (0.310, 1.022)	360 VS 270, HR 0.343 ( 0.185, 0.636)
Wenzhe Fan 2019	NA	NA	500, twice daily	mRECIST:24% VS 4%	59% VS 14%	0% VS 0%	24% VS 4%	26% VS 10%	35% VS 89%	183 VS 111, HR 0.61( 0.48, 0.77)	360 VS 210, HR 0.443 (0.306, 0.641)
Xuefeng Kan 2020	NA	NA	500, twice daily	mRECIST:51% VS 10%	59% VS 33%	4% VS 0%	47% VS 10%	8% VS 23%	41% VS 67%	210 VS 90	390 VS 240, HR 0.35 ( 0.26, 0.49)
Juanfang Liu 2019	11.8% VS 8.3%	NA	500, twice daily	mRECIST: 55.9% vs 31.3%	70.6% vs 43.8%	0% VS 0%	55.9% VS 31.2%	14.7% VS 12.5%	29.4% VS 56.3%	NA	210 VS 167, HR 0.346 (0.203, 0.591)
Yuanyuan Li 2021	NA	NA	500, twice daily	mRECIST:4.76% VS 40.74%	23.81% VS 77.78%	0%VS 0%	4.8% VS 40.7%	19% VS 37.0%	76.2% VS 22,2%	NA	324 VS 399, HR 0.455 (0.245, 0.848)
Zhiyu Qiu 2019	NA	NA	500, twice daily	RECIST: 16.7% VS 8.4%	81.0% VS 53.0%	4.8% VS 3.6%	11.9% VS 4.8%	64.3% VS 44.6%	19.0% VS 47.0%	NA	510 VS 321, HR 0.28 (0.158, 0.499)
Lujun Shen 2020	NA	NA	500, twice daily	NA	NA	NA	NA	NA	NA	NA	546 VS 255, HR 0.38 ( 0.22, 0.66)
Masatoshi Kudo 2014	16% VS 15%	NA	800, once-daily	mRECIST:48% VS 42%	79% VS 79%	22% VS 11%	26% VS 31%	31% VS 37%	9% VS 18%	NA	792 VS 783, HR 0.9 (0.66, 1.23)
Zhigang Fu 2021	NA	NA	12 mg (≥ 60 kg) or 8 mg (< 60 kg) once daily based on body weight/0, once-daily	mRECIST: 68.3% VS 31.7%	93.3% VS 86.7%	10.0% VS 5.0%	58.3% VS 26.7%	25.0% VS 55.0%	6.7% VS 13.3%	NA	NA, HR 0.466 (0.226, 0.886)
Tim Meyer 2017	34% VS 33%	B + C + alcohol: 2% VS 2% B + alcohol: 2% VS 2%	400, twice daily	mRECIST: 54% VS 52%	mRECIST: 75% VS 77%	mRECIST: 29% VS 23%	mRECIST: 25% VS 29%	mRECIST: 21% VS 25%	mRECIST: 8% VS 10%	326 VS 320, HR 0.88 (0.67,1.17)	631 VS 598, HR 0.91 (0.67, 1.24)
Xuesong Yao 2016	NA	NA	400, twice daily	mRECIST: 8% VS 1%	32% VS 24%	0% VS 0%	8% VS 1%	24% VS 23%	68% VS 76%	306 VS 201	651 VS 345, HR 0.481 (0.297, 0.778)
Riccardo Lencioni 2016	17.5% VS 19.6%	B + alcohol: 1.9% VS 0.7% C + alcohol: 1.9% VS 2%	400, twice daily	mRECIST: 42.9% VS 34.6%	80.5% VS 71.9%	13.6% VS 13.1%	29.2% VS 21.6%	37.7% VS 37.3%	10.4% VS 19.6%	169 VS 166, HR 0.797 (0.588, 1.08)	270 VS 272, HR 0.898 (0.606, 1.330)
Masatoshi Kudo 2019	NA	NA	400, twice daily	RECICL: 71.3% VS 61.8%	83.8% VS 77.6%	28.8% VS 27.6%	42.5% VS 34.2%	12.5% VS 15.8%	2.5% VS 3.9%	801 VS 492, HR 0.54 (0.35, 0.83)	NA
Zhexuan Wang 2020	NA	NA	400, twice daily	NA	NA	NA	NA	NA	NA	219 VS 189, HR 0.75 (0.60, 0.93)	672 VS 666, HR 0.87 (0.74,1.02)
Kangshun Zhu 2014	NA	NA	400, twice daily	mRECIST: 28.3% VS 4.4%	57% VS 13%	0% VS 0%	28.3% VS 4.4%	28.3% VS 8.9%	43.5% VS 86.7%	180 VS 90	330 VS 180, HR 0.429 (0.268, 0.690)
Masatoshi Kudo 2011	8.2% VS 5.2%	NA	400, twice daily	NA	NA	NA	NA	NA	NA	162 VS 111, HR 0.87( 0.7, 1.09)	NA, HR 1.06 (0.69, 1.64)
Yan Zhao 2016	NA	NA	400, twice daily	NA	NA	NA	NA	NA	NA	393 VS 150	669 VS 537, HR 0.4(0.4, 0.83)
Katrin Hoffmann 2015	29.1% VS 42.3%	NA	400, twice daily	mRECIST: 20.8%: 26.9%	66.7% VS 73.1%	4.3% VS 0%	17.4% VS 26.9%	47.8% VS 46.2%	30.4% VS 26.9%	71 VS 85, HR 1.106 (0.387, 3.162)	NA
Jianbing Wu 2017	NA	NA	400, twice daily	mRECIST:NA	73.4% VS 51.6%	NA	16.7% VS 6.5%	56.7% VS 45.1%	26.6% VS 48.4%	279 VS 102,	537 VS 213, HR 0.151 (0.071, 0.322)
Hao Hu 2014	NA	NA	400, twice daily	NA	NA	NA	NA	NA	NA	78 VS 57 HR 0.62 (0.47,0.82)	210 VS147, HR 0.63 (0.48, 0.84)
Wei Bai 2013	NA	NA	400, twice daily	RECIST: 9.7% VS 3.4%	58.5% VS 44.5%	0% VS 0%	9.7% VS 3.4%	48.8% VS 41.1%	41.5% VS 55.5%	189 vs 129, HR 0.6 (0.422, 0.853)	225 vs 153, HR 0.61(0.42, 0.884)
Zhenwei Peng 2019	3.9% VS 3.8%	NA	400, twice daily	mRECIST: 72.3% VS 50.0%	87.3% VS 80.6%	34.5% VS 20.8%	38.1% VS 29.2%	14.5% VS 30.6%	NA	NA	516 VS 363, HR 0.62(0.44, 0.89)
Baosheng Ren 2019	NA	NA	400, twice daily	NA	NA	NA	NA	NA	NA	NA	870 ± 216 VS 447 ± 45, HR 0.684 (0.470,0.997)
Xinhua Zou 2021	NA	NA	400, twice daily	mRECIST: 23.81% VS 16.28%	80.95% VS 55.81%	4.76% VS 0.00%	19.05% VS 16.28%	57.14% VS 39.53%	19.05% VS 44.19%	NA	960 VS 630, HR 0.6155 (0.3978, 0.9524)
Xue-Fen Lei 2018	NA	NA	400, twice daily	mRECIST: 60.5% VS 41.4%	86.8% VS 65.5%	31.6% VS 13.8%	28.9% VS 27.6%	26.3% VS 24.1%	13.2% VS 34.5%	NA	1056 VS 660, HR 0.113 (0.036, 0.350)
Takamasa Ohki 2015	NA	NA	400, twice daily	NA	NA	NA	NA	NA	NA	NA	861 VS 467, HR 0.43 (0.24, 0.76)
Xuying Wan 2016	NA	NA	400, twice daily	NA	NA	NA	NA	NA	NA	NA	607 VS 419, HR 0.76 (0.61, 0.94)

*RECIST* Response Evaluation Criteria In Solid Tumors, *mRECIST* modified RECIST, *TACE* transarterial chemoembolization, *DEB-TACE* drug-eluting bead transarterial chemoembolization, *BCLC* The Barcelona Clinic Liver Cancer, *ECOG* Eastern Cooperative Oncology Group, *NA* not available, *RCT* randomized controlled trial, *Child–Pugh* Child–Turcotte–Pugh, *ORR* objective response rate, *DCR* disease control rate, *CR* complete response, *PR* partial response, *SD* stable disease, *PD* progressive disease

## Meta-analysis

### Time to progression

**Fig. 3:**
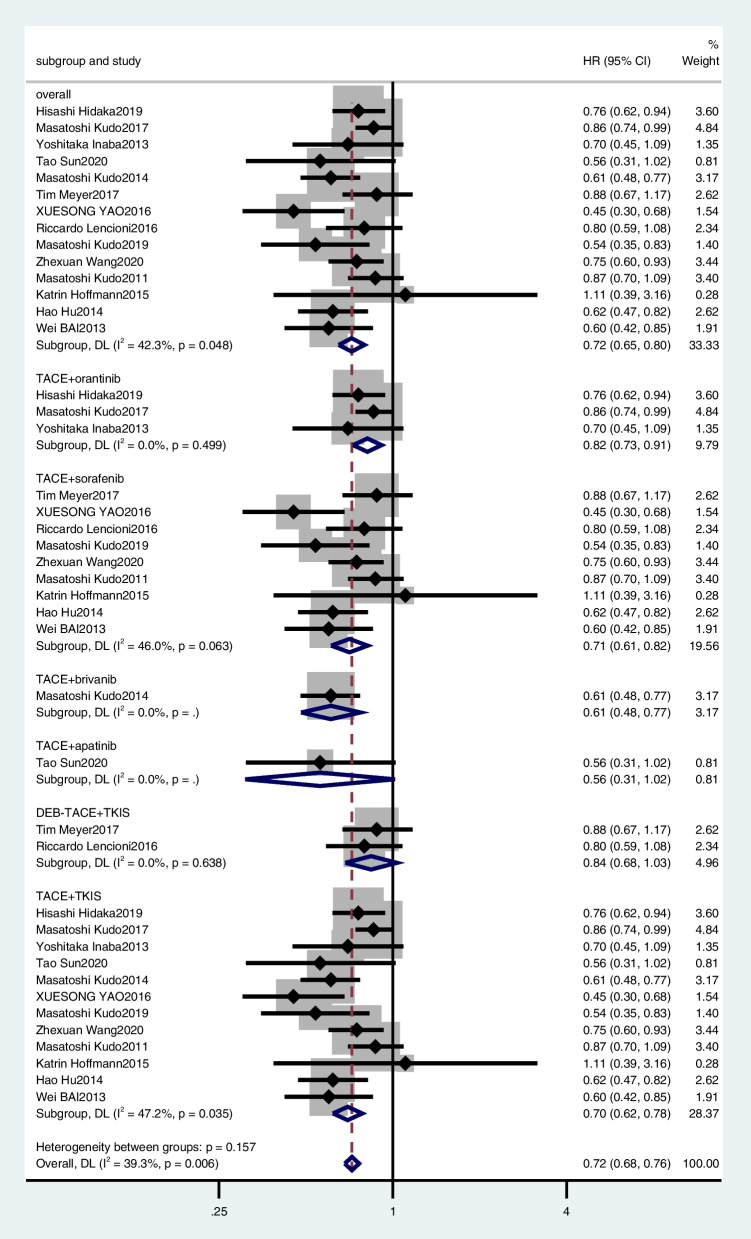
Meta-analysis for treatment effects of TKIS in combination with TACE on time to progression (TTP) in patients with unresectable hepatocellular carcinoma

Sixteen [19, 27–32, 39, 41, 43, 46–51] studies were excluded from the TTP meta-analysis because it did not provide HR and 95% CI for TTP. Thus, 14 studies were included in the meta-analysis [18, 24–26, 33–38, 40, 42, 44, 45]. The random-effects model analysis results were as follows: TACE plus TKIs had a better outcome with TTP than TACE plus placebo or TACE alone, with a combined HR of 0.72 (95% CI, 0.65–0.80) (Fig. 3).

In the subgroup analyses, TACE plus orantinib (combined HR, 0.82; 95% CI, 0.73–0.91) (Fig. 3), TACE plus sorafenib (combined HR, 0.71; 95% CI, 0.61–0.82) (Fig. 3), only one article was included in TACE plus apatinib (HR, 0.560; 95% CI, 0.310–1.022), and TACE plus brivanib (HR, 0.61; 95% CI, 0.48–0.77), so the combined HR was no longer used in the analysis. DEB-TACE plus TKIS (combined HR, 0.84, 95%CI, 0.68–1.03), TACE plus TKIS (combined HR, 0.70, 95%CI, 0.62–0.78).

**Fig. 4:**
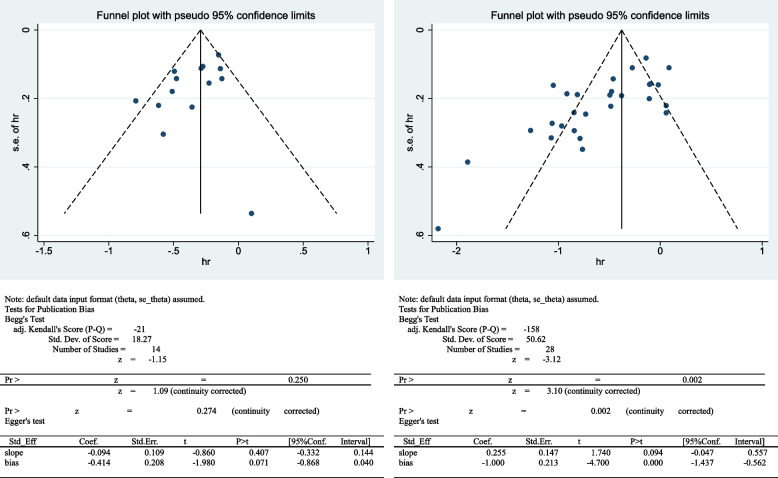
Funnel plot for random effects meta analysis of mean difference in TTP and OS

A sensitivity analysis was performed to investigate whether the results were stable. We recalculated the summary HR by excluding each individual study. The range of the combined HR was from 0.70 (95% CI, 0.63–0.78) to 0.73 (95% CI, 0.66–0.81) when the Yao 2016 et al. study was excluded. The heterogeneity was also significantly reduced from 42.3 to 27.4%. The results showed that no individual study significantly affected the pooled effect size. Funnel plots and Egger’s test (*t* =  − 1.98, *p* = 0.071) showed no evidence of publication bias (Fig. 4).

### Overall survival

**Fig. 5:**
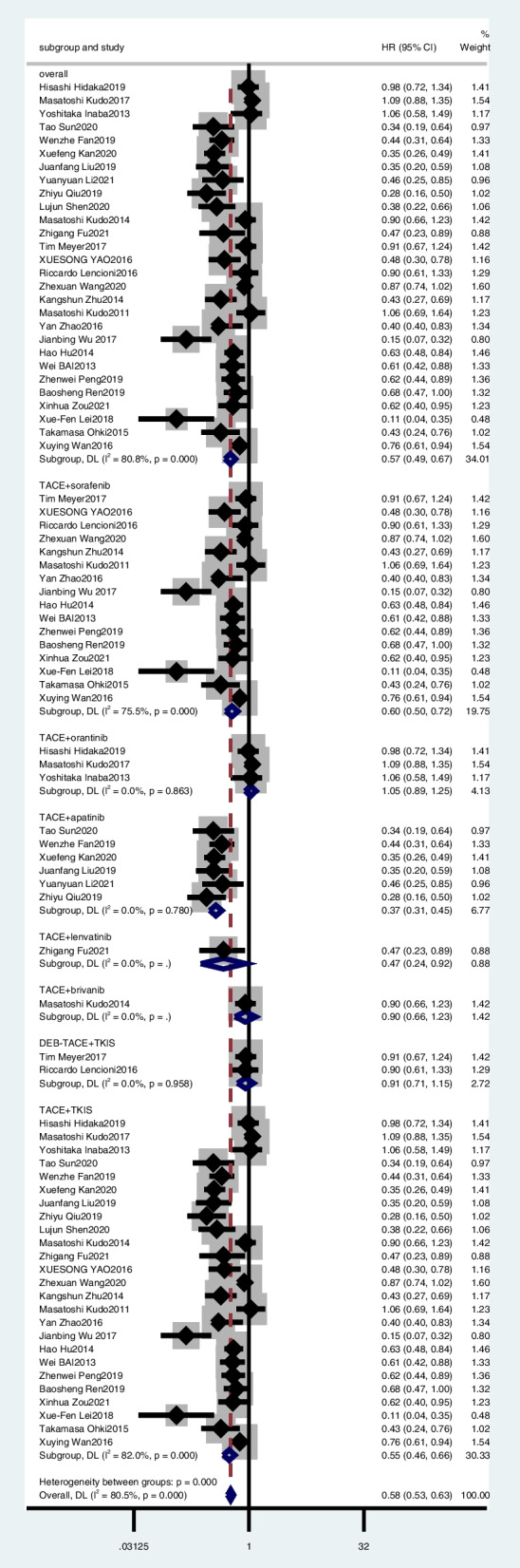
Meta-analysis for treatment effects of TKIS in combination with TACE on overall survival (OS) in patients with unresectable hepatocellular carcinoma

Of the 30 studies, 28 [18, 19, 24–41, 43–51] provided HR and 95% CI for OS. The pooled results showed that TACE plus TKIs was significantly associated with better OS (combined HR, 0.57; 95% CI, 0.49–0.67) (Fig. 5).

In the subgroup analysis, TACE plus orantinib (combined HR, 1.05; 95% CI, 0.89–1.25) (Fig. 5), TACE plus apatinib (combined HR, 0.37; 95% CI, 0.3–0.44) (Fig. 5), and TACE plus sorafenib (combined HR, 0.60; 95% CI, 0.50–0.72) (Fig. 5). However, TACE plus brivanib (HR, 0.90; 95% CI, 0.66–1.23) and TACE plus lenvatinib (HR, 0.466; 95% CI, 0.226–0.886) were reported by only one article, so the HR values were not combined separately. DEB-TACE plus TKIS (combined HR, 0.91, 95%CI, 0.71–1.15), TACE plus TKIS (combined HR, 0.55, 95%CI, 0.46–0.66).

We performed a sensitivity analysis to explore the robustness of our analysis and recalculated the pooled HR by excluding each individual study. The range of the combined HR was from 0.56 (95% CI, 0.48–0.66) to 0.59 (95% CI, 0.50–0.69). The results showed that no individual study significantly affected the pooled effect size. Funnel plots and Egger’s test (*t* =  − 4.700, *p* < 0.05) showed publication bias (Fig. 4).

### Adverse effects

**Fig. 6:**
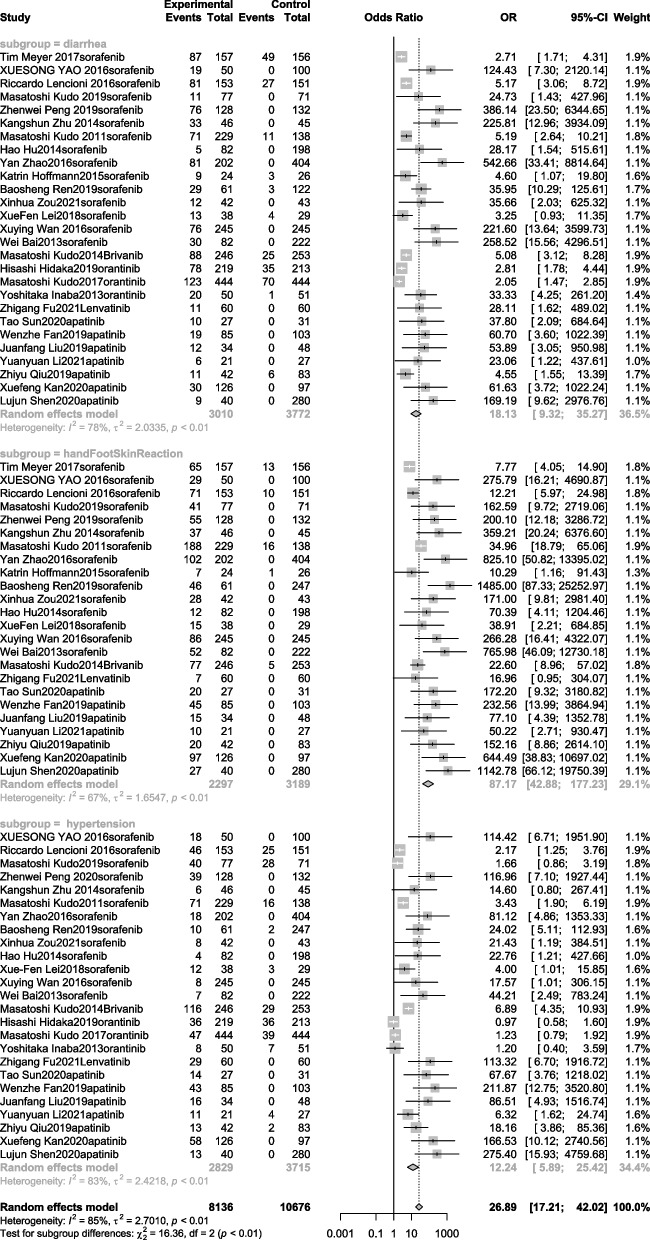
Meta-analysis for treatment effects of TKIS in combination with TACE on adverse events (hand-foot skin reaction, hypertension, diarrhea) in patients with unresectable hepatocellular carcinoma

The AEs are summarized in Additional file 3: Table 4. AEs were classified according to the Common Terminology Standard for Adverse Events (version 4.03) [52]. In the random-effects model for adverse reactions of hand-foot skin reaction, diarrhea, and hypertension, the most common AEs in studies related to TACE plus TKIs treatment were hand and foot skin reactions (OR, 87.17; 95% CI, 42.88–177.23), diarrhea (OR, 18.13; 95% CI, 9.32–35.27), and hypertension (OR, 12.24; 95% CI, 5.89–25.42). The forest plot results are shown in Fig. 6.

The common adverse reactions to TACE plus sorafenib were hand-foot skin reactions, diarrhea, hypertension, hair loss, and bleeding. Common adverse reactions to TACE plus brivanib included hand-foot skin reactions, hypertension, rash/desquamation, nausea, and fever. Common adverse reactions to TACE plus orantinib included diarrhea, gastrointestinal disease, abdominal pain, elevated alanine transaminase (ALT) levels, and fever. Common adverse reactions to TACE plus lenvatinib included diarrhea, nausea, hypertension, gastric ulcers, and bleeding. Common adverse reactions to TACE plus apatinib included diarrhea, gastric ulcers, hemorrhage, erythema multiforme, and hypoalbuminemia.

### Tumor response rates

**Fig. 7:**
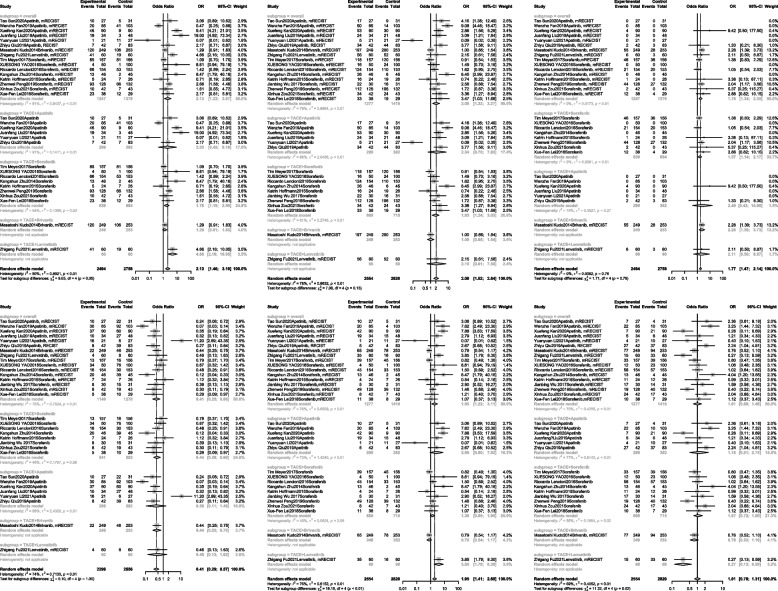
Meta-analysis for treatment effects of TKIS in combination with TACE on tumor response rates in patients with unresectable hepatocellular carcinoma

Seventeen of the 30 studies [19, 26–31, 33–36, 39, 42, 43, 46, 48, 49] were used to analyze the tumor response rates. The ORR, DCR, CR, PR, SD, and PD were evaluated and described according to modified Response Evaluation Criteria in Solid Tumors [53, 54], ORR (OR, 2.13; 95% CI, 1.23–3.67), DCR (OR, 2.08; 95% CI, 1.32–3.67), CR (OR, 1.78; 95% CI, 1.34–2.35), PR (OR, 1.95; 95% CI, 1.22–3.11), PD (OR, 0.41; 95% CI, 0.25–0.66) were better in the combined therapy versus TACE alone group. However, no significant difference was observed in SD (OR, 1.01; 95% CI, 0.69–1.48) (Fig. 7).

In the subgroup analysis, TACE plus apatinib treatment showed no significant difference between the experimental and control groups in ORR (OR, 2.03; 95% CI, 0.45–9.16), DCR (OR, 2.34; 95% CI, 0.70–7.83), CR (OR, 2.49; 95% CI, 0.42–14.9), PR (OR, 2.68; 95% CI, 0.90–7.92), PD (OR, 0.39; 95% CI, 0.11–1.46), SD (OR, 1.18; 95% CI, 0.51–2.74). For TACE plus sorafenib, TACE plus TKIs was better than TACE alone or TACE plus placebo in terms of ORR (OR, 1.78; 95% CI, 1.19–2.66), DCR (OR, 1.93; 95% CI, 1.24–3.03), CR (OR, 1.57; 95% CI, 1.14–2.17), and PD (OR, 0.44; 95% CI, 0.28–0.69). However, no significant differences were observed in PR (OR, 1.36; 95% CI, 0.95–1.95) and SD (OR, 1.07; 95% CI, 0.72–1.60) (Fig. 7). Moreover, TACE plus brivanib and TACE plus lenvatinib were reported in only one article; therefore, the OR values were not combined separately.

### Sensitivity analysis

**Fig. 8:**
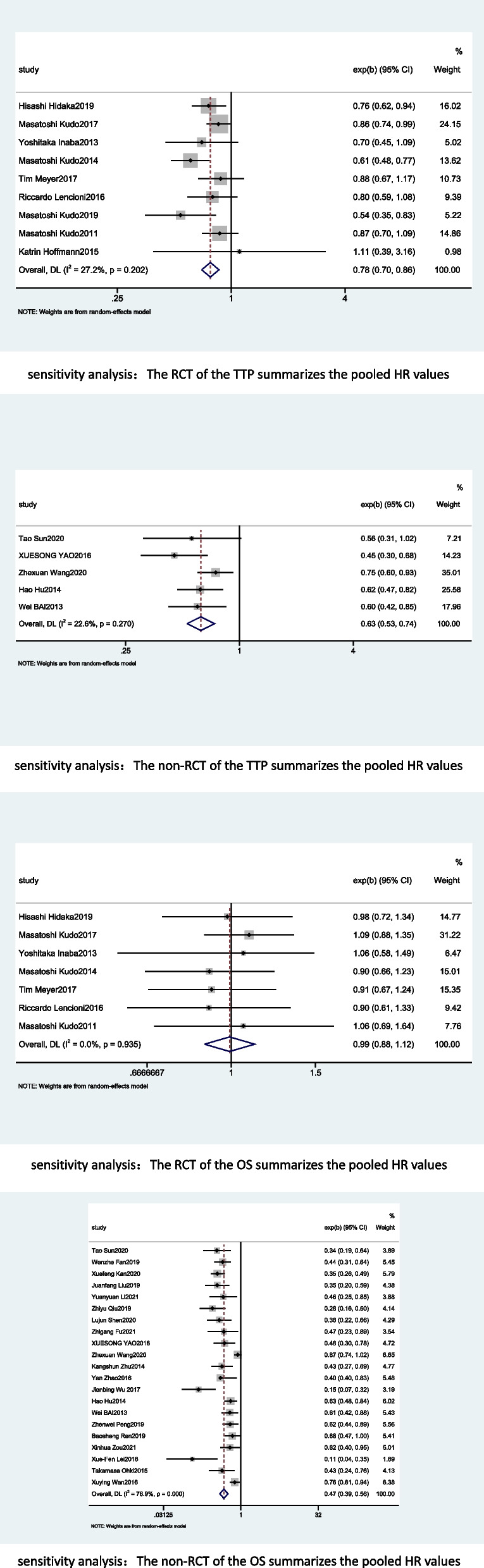
Sensitivity analysis for treatment effects of TKIS in combination with TACE on TTP and OS

Sensitivity analyses were performed for TTP and OS. In terms of TTP, according to article type, we divided the included studies into RCTs and non-RCTs and combined their HR values. Nine RCTs [18, 24, 25, 33, 34, 36, 37, 40, 42] had a combined HR of 0.78, 95% CI of 0.70–0.86,* I*^*2*^ = 27.2% and moderate certainty evidence, while 5 non-RCTs [26, 35, 38, 44, 45] had a combined HR of 0.63, 95% CI of 0.53–0.74, and *I*^*2*^ = 22.6%, and heterogeneity was also significantly reduced. Similarly, in terms of OS, 7 RCTs [18, 24, 25, 33, 34, 36, 40] had a combined HR of 0.99, 95% CI of 0.88–1.12, *I*^*2*^ = 0.0% and moderate certainty evidence, while 21 non-RCTs [19, 26–32, 35, 37–39, 41, 43–51] had a combined HR of 0.41, 95% CI of 0.39–0.56, and *I*^*2*^ = 76.9%. Details of the forest plots are shown in Fig. 8.

## Discussion

This meta-analysis enrolled nine RCTs and 21 observational studies with a total of 8246 patients. The results indicated that TACE plus TKIs has an advantage over TACE alone or TACE plus placebo in terms of OS and TTP with an acceptable AE rate. Common AEs associated with TACE plus TKIs therapy mainly include hand-foot skin reaction, diarrhea, hypertension, fatigue, nausea, abdominal pain, vomiting, elevated ALT, fever, and voice change.

Clinically, TACE has a high cost performance because its mini-invasion and excision characteristics are similar to those of surgery. It also has the limitation that a single-pass treatment rarely removes all of the live tumor, and the remaining part can regenerate blood vessels and acquire stronger invasion and metastasis ability in an anaerobic environment. TKIs, a common treatment for advanced uHCC, are usually administered orally, with good patient compliance and slight AEs. Moreover, TKIs can also suppress angiogenesis. To some extent, this seems to compensate for the shortcomings of TACE, a local treatment that can reduce tumor burden, while TKIs are a systemic treatment that can control the disease as a whole. Evidently, the combination of topical and systemic remedies plays a complementary role and provides patients with more benefits.

Several previous meta-analyses evaluated TACE plus sorafenib in patients with uHCC [55–60]. The results of TACE plus sorafenib prolonged TPP in uHCC patients, consistent with our conclusion. This further proves that TACE plus TKIs has a synergistic effect in the treatment of uHCC. However, the results of the five [25, 34, 36, 40, 42] studies we included showed that TACE plus TKIs did not prolong TTP in uHCC. Among them, two [25, 40] studies in Asia indicated that TACE plus TKIs tended to prolong TTP, but this result was not statistically significant. The three [34, 36, 42] studies included more Europeans, and the results showed that combination therapy did not improve TTP in uHCC patients. Interestingly, most of the patients from Asia [25, 40] had hepatitis B or C and most had a Child–Pugh grade of A, while most of the patients included in Europe had alcoholic hepatitis and had a Child–Pugh grade of A. This phenomenon deserves attention since, compared with Europeans, Asians have better results with combination therapy. Most Asian liver cancers are caused by hepatitis B virus infection and are more likely to be treated with TACE combined with TKIs therapy; this may require further research.

Moreover, the Hoffmann 2015 [42] study showed that TACE combined with sorafenib is not suitable for the treatment of HCC patients before liver transplantation. In terms of OS, our study results showed that TACE plus TKIs may prolong OS in patients with uHCC. However, the included studies of TACE plus orantinib, TACE plus brivanib, and TACE plus sorafenib could not prolong OS in uHCC patients, indicating that TACE plus orantinib and TACE plus brivanib after TACE treatment may be a coincidence, but there is a chance that the order of the therapy contributes to the final efficacy. At the same time, the poor treatment effect of TACE plus sorafenib may be caused by the smaller dosage and shorter administration time in the experimental group than in the control group. In terms of tumor response rates, our meta-analysis also demonstrated that TACE plus TKIs had significantly better ORR and DCR. This may be due to the cytotoxic effect of TACE as adjuvant therapy with TKIs. In terms of AEs, our study also showed that the morbidity rate was much higher in the combination treatment group, and the complications were mostly TKI-related. These results are consistent with those of a previous study [61], which showed that hand-foot skin reactions, diarrhea, and hypertension were the most common, indicating that compared with TKIs alone or TACE plus placebo, although the incidence of AEs increased with TACE plus TKIs, there were no unbearable AEs, which were all within the acceptable range.

In terms of TTP, subgroup analysis of included studies according to the type of TACE combination drug showed that, compared to TACE alone, TACE plus orantinib, TACE plus sorafenib, TACE plus brivanib, and TACE plus apatinib all support the idea that TACE plus TKIs are more likely to improve TTP in patients with uHCC. In addition, a sensitivity analysis of the included studies according to study type, the combined HR of nine RCTs [18, 24, 25, 33, 34, 36, 37, 40, 42] and five non-RCTs [26, 35, 38, 44, 45], all illustrate that TACE plus TKIs are more able to prolong TTP in uHCC, demonstrating the stability of our results. In terms of tumor response rates, a subgroup analysis of the included studies according to TACE combination drug, the results demonstrate that, compared to TACE alone, TACE plus apatinib and TACE plus brivanib did not improve ORR and DCR in uHCC patients. However, TACE plus sorafenib and TACE plus lenvatinib resulted in significantly better ORR and DCR. Subgroup analyses according to conventional and drug-eluting beads showed that DEB-TACE did not show superiority over conventional TACE in terms of TTP and OS, but it minimizes systemic toxicity and provides a standardized embolic effect. So it still provides another option for clinicians. Moreover, few studies of DEB-TACE were included in our study, and we hope that more and more comprehensive studies will be conducted in the future.

In terms of OS, a subgroup analysis was performed according to TACE combination drug, and the results indicated that TACE plus orantinib combined HR showed that combination therapy did not improve OS compared to TACE alone. However, TACE plus apatinib, TACE plus sorafenib, TACE plus brivanib, and TACE plus lenvatinib showed that the combination therapy significantly improved the OS of uHCC compared with TACE alone. A sensitivity analysis of the included studies according to study type and the results between RCT and non-RCT-combined HR showed opposite results. The results of seven RCTs [18, 24, 25, 33, 34, 36, 40] combined with HR showed that TACE plus TKIs treatment did not prolong the OS of uHCC patients, and with the *I*^*2*^ = 0, the heterogeneity was low. The seven RCTs, including three of TACE plus orantinib, one of TACE plus brivanib, and three of TACE plus sorafenib with a total sample size of 3002 (37%), were conducted in Japan and Korea. Interestingly, the combined HR of 21 non-RCT studies [19, 26–32, 35, 37–39, 41, 43–51] showed that the combination therapy could prolong OS in patients with uHCC better than TACE alone. Among the 21 RCTs, 20 studies were conducted in China and one was conducted in Japan, with a sample size of 5244 (63%). Perhaps this is a coincidence, but it cannot be ruled out that TACE plus TKIs may be more effective in Chinese patients with prolonged OS. None of the included studies of TACE plus orantinib and TACE plus brivanib support the advantage of combination therapy in prolonging OS in patients with uHCC over TACE alone. Orantinib and brivanib are very good clinical drugs, and their combined treatment with TACE requires additional studies.

Our study has the following strengths. Firstly, it is the first to systematically evaluate the clinical benefits and risks of TACE plus TKIs. Second, it provides more options and evidence to support the clinical treatment of uHCC with TACE plus TKIs. Third, we used rigorous methodological criteria and conducted systematic searches of large sample sizes and in-depth analyses of different subgroups. It also has the following limitations. First, the population characteristics of the included trials (age, etiology of liver disease, vascular invasion, and previous treatment), TKI regimen (treatment lag, treatment duration, treatment sequence, number of prior TACE courses, and dose administered), and study designs vary widely, which may increase heterogeneity and affect the results. Secondly, there are differences chemotherapy agents in TACE with different embolic and drug-eluting bead (e.g., size or type) in different stuies, and these factors may affect the pooled result. Thirdly, the small sample size of some of the included studies may lead to overestimation of the treatment effect.

In conclusion, the current meta-analysis showed that TACE plus TKIs can significantly improve TTP and OS in patients with uHCC with tolerable toxicity. Based on patient specificity, TKIs is a more flexible option for the treatment of uHCC. Owing to the accumulation of new evidence, making the overall situation closer to the real situation, TACE plus TKIs may be a better choice for treating uHCC. We hope that more high-quality studies will be conducted to further support our conclusions.

## Supplementary Information

**Additional file 1:** **Table 1** Assessment of retrospective cohort studies using Newcastle–Ottawa scale.

**Additional file 2:** **Table 2** Grade evidence profile of TTP, OS, adverse event and tumor response rates. Certainty of evidence and summary effect estimates assessed byGRADE (grading of recommendations, assessment, development, and evaluation) of randomized controlled trials.

**Additional file 3:** **Table 4** Summary of adverse events.

## Data Availability

We confirm that we have given due consideration to the protection of intellectual property associated with this work and that there are no impediments to publication, including the timing of publication, with respect to intellectual property. In so doing, we confirm that we have followed the regulations of our institutions concerning intellectual property. We understand that the corresponding author is the sole contact for the editorial process (including Editorial Manager and direct communications with the office). He is responsible for communicating with the other authors about progress, submissions of revisions and final approval of proofs. We confirm that we have provided a current, correct email address which is accessible by the corresponding author and which has been configured to accept email from 104,220,421@qq.com.
