# Supplementary material for: Transarterial chemoembolization (TACE) plus tyrosine kinase inhibitors versus TACE in patients with hepatocellular carcinoma: a systematic review and meta-analysis
Source: World J Surg Oncol. 2023 Mar 31;21:120. doi: 10.1186/s12957-023-02961-7 (PMC10064711; doi:10.1186/s12957-023-02961-7)
Supplement: Supplementary file 1 — Additional file 1: Table 1 Assessment of retrospective cohort studies using Newcastle–Ottawa scale. [file 12957_2023_2961_MOESM1_ESM.docx]

| Assessment of retrospective cohort studies using Newcastle–Ottawa scale | | | | |
| --- | --- | --- | --- | --- |
| Author/year | Selection(4 score) | Comparability(2 score) | Outcome(3 score) | Total(9 score) |
| Wenzhe Fan(2019) | ✱✱✱ | ✱✱ | ✱✱ | 7 |
| Lujun Shen(2020) | ✱✱✱ | ✱✱ | ✱✱ | 7 |
| Yuanyuan Li(2021) | ✱✱✱ | ✱✱ | ✱✱ | 7 |
| Xuefeng Kan（2020） | ✱✱✱ | ✱✱ | ✱✱ | 7 |
| Juanfang Liu(2019) | ✱✱✱ | ✱✱ | ✱✱ | 7 |
| Tao Sun(2020) | ✱✱✱ | ✱✱ | ✱✱ | 7 |
| XUESONG YAO(2016) | ✱✱✱ | ✱✱ | ✱✱ | 7 |
| Kangshun Zhu（2014) | ✱✱✱ | ✱✱ | ✱✱ | 7 |
| Yan Zhao（2016) | ✱✱✱ | ✱✱ | ✱✱ | 7 |
| Jianbing Wu (2017) | ✱✱ | ✱✱ | ✱✱ | 6 |
| Baosheng Ren(2019) | ✱✱✱ | ✱✱ | ✱✱ | 7 |
| Xinhua Zou(2021) | ✱✱ | ✱✱ | ✱✱ | 6 |
| Takamasa Ohki（2015） | ✱✱ | ✱✱ | ✱✱ | 6 |
| Xuying Wan(2016) | ✱✱ | ✱✱ | ✱✱ | 6 |
| Zhexuan Wang（2020) | ✱✱✱ | ✱✱ | ✱✱ | 7 |
| Wei BAI（2013） | ✱✱✱ | ✱✱ | ✱✱ | 7 |
| Xue-Fen Lei(2018) | ✱✱✱ | ✱✱ | ✱✱ | 7 |
| Hao Hu(2014) | ✱✱✱✱ | ✱✱ | ✱✱ | 8 |
| Zhenwei Peng（2019） | ✱✱✱✱ | ✱✱ | ✱✱✱ | 9 |
| Zhiyu Qiu(2019) | ✱✱✱ | ✱✱ | ✱✱ | 7 |
| Zhigang Fu(2021） | ✱✱✱ | ✱✱ | ✱✱ | 7 |
