# Supplementary material for: Transarterial chemoembolization (TACE) plus tyrosine kinase inhibitors versus TACE in patients with hepatocellular carcinoma: a systematic review and meta-analysis
Source: World J Surg Oncol. 2023 Mar 31;21:120. doi: 10.1186/s12957-023-02961-7 (PMC10064711; doi:10.1186/s12957-023-02961-7)
Supplement: Supplementary file 2 — Additional file 2: Table 2 Grade evidence profile of TTP, OS, adverse event and tumor response rates. Certainty of evidence and summary effect estimates assessed byGRADE (grading of recommendations, assessment, development, and evaluation) of randomized controlled trials. [file 12957_2023_2961_MOESM2_ESM.pdf]

Author(s):
Question: Table 4 Grade evidence profile of TTP, OS, adverse event and tumor response rates. Certainty of evidence and summary effect estimates assessed by GRADE (grading of recommendations, assessment, development, and evaluation) of randomised controlled trials.
Setting:
Bibliography:

Certainty assessment							№ of patients		Effect		Certainty	Importance
**№ of studies**	**Study design**	**Risk of bias**	**Inconsistency**	**Indirectness**	**Imprecision**	**Other considerations**	**TACE+TKIS**	**TACE**	**Relative (95% CI)**	**Absolute (95% CI)**		
**TTP**												
9	randomised trials	serious[a]	not serious	not serious	not serious	none	1607 participants<br>-	1601 participants<br>0.0%	**HR 0.00** (0.70 to 0.86) [TTP]		⊕⊕⊕◯ Moderate	
**OS**												
7	randomised trials	not serious	not serious	not serious	serious[b]	none	1503 participants<br>-	1499 participants<br>0.0%	**HR 0.00** (0.88 to 1.12) [OS]		⊕⊕⊕◯ Moderate	
**Adverse event-diarrhea**												
9	randomised trials	serious[a]	serious[c]	not serious	not serious	none	568/1599 (35.5%)	221/1503 (14.7%)	**OR 0.00** (2.69 to 5.40)	**-- per 1,000** (from 170 more to 335 more)	⊕⊕◯◯ Low	
**Adverse event-hand foot skin reaction**												
6	randomised trials	serious[d]	serious[e]	not serious	not serious	none	449/886 (50.7%)	45/795 (5.7%)	**OR 0.00** (9.30 to 33.32)	**-- per 1,000** (from 302 more to 610 more)	⊕⊕◯◯ Low	
**Adverse event-hypertension**												
7	randomised trials	serious[f]	serious[g]	not serious	not serious	none	364/1418 (25.7%)	180/1321 (13.6%)	**OR 0.00** (1.19 to 3.49)	**-- per 1,000** (from 22 more to 219 more)	⊕⊕◯◯ Low	
**CR**												
4	randomised trials	serious[h]	not serious	not serious	serious[i]	none	123/584 (21.1%)	84/588 (14.3%)	**OR 0.00** (1.02 to 2.40)	**-- per 1,000** (from 2 more to 143 more)	⊕⊕◯◯ Low	
**DCR**												
4	randomised trials	serious[h]	not serious	not serious	serious[i]	none	455/584 (77.9%)	449/588 (76.4%)	**OR 0.00** (0.81 to 1.46)	**-- per 1,000** (from 40 fewer to 61 more)	⊕⊕◯◯ Low	
**ORR**												
4	randomised trials	serious[h]	not serious	not serious	serious[i]	none	276/584 (47.3%)	202/588 (34.4%)	**OR 0.00** (0.66 to 1.08)	**-- per 1,000** (from 87 fewer to 18 more)	⊕⊕◯◯ Low	
**PD**												
4	randomised trials	serious[h]	not serious	not serious	not serious	none	58/584 (9.9%)	99/588 (16.8%)	**OR 0.00** (0.38 to 0.78)	**-- per 1,000** (from 97 fewer to 32 fewer)	⊕⊕⊕◯ Moderate	
**PR**												
4	randomised trials	serious[h]	not serious	not serious	serious[i]	none	153/584 (26.2%)	163/588 (27.7%)	**OR 0.00** (0.65 to 1.34)	**-- per 1,000** (from 78 fewer to 62 more)	⊕⊕◯◯ Low	
**SD**												
4	randomised trials	serious[h]	not serious	not serious	not serious	none	179/584 (30.7%)	202/588 (34.4%)	**OR 0.00** (0.66 to 1.08)	**-- per 1,000** (from 87 fewer to 18 more)	⊕⊕⊕◯ Moderate	

CI: confidence interval; **HR:** hazard Ratio; **OR:** odds ratio

Explanations

a. 9 studies were included and 5 studies were some concern risk of bias.

b. 95% CIs around the pooled included no effect and appreciable harm
c. Heterogeneity:I2=66%,P<0.001.
d. 4 studies were included and 6 studies were some concern risk of bias.
e. Heterogeneity:I2=66%,P=0.01.
f. 4 studies were included and 7 studies were some concern risk of bias.
g. Heterogeneity:I2=86%,P<0.01
h. 2 studies were included and 4 studies were some concern risk of bias.
i. 95%CI around the pooled included no effect and appreciable benefit.
