# Supplementary material for: Transarterial chemoembolization (TACE) plus tyrosine kinase inhibitors versus TACE in patients with hepatocellular carcinoma: a systematic review and meta-analysis
Source: World J Surg Oncol. 2023 Mar 31;21:120. doi: 10.1186/s12957-023-02961-7 (PMC10064711; doi:10.1186/s12957-023-02961-7)
Supplement: Supplementary file 3 — Additional file 3: Table 4 Summary of adverse events. [file 12957_2023_2961_MOESM3_ESM.docx]

|  |  | 1st AU (year) | Tim Meyer2017  (DEB-TACE+ sorafenib  VS  DEB-TACE+ placebo) | XUESONG YAO2016  (TACE+sorafenib  VS TACE+alone) | Riccardo Lencioni2016  (DEB-TACE + sorafenib VS  DEB-TACE + placebo) | Masatoshi Kudo2019  (TACE+sorafenib VS  TACE+ alone ) | Zhexuan Wang2020 | Zhenwei Peng2019  (TACE+sorafenib  VS  TACE) | Kangshun Zhu2014  (TACE+sorafenib VS  TACE) | Masatoshi Kudo2011  (TACE+sorafenib  VS TACE + placebo) | Yan Zhao2016  (TACE+sorafenib  VS  TACE) | Katrin Hoffmann2015  (TACE+sorafenibVS  TACE  + placebo) | Baosheng Ren2019  (TACE+sorafenib VS  TACE) | Jianbing Wu 2017 | Xinhua Zou2021  (TACE+sorafenib  VS  TACE) | Hao Hu2014  (TACE+sorafenib  VS  TACE) | Xue-Fen Lei2018  (TACE+sorafenib  VS  TACE) |
| --- | --- | --- | --- | --- | --- | --- | --- | --- | --- | --- | --- | --- | --- | --- | --- | --- | --- |
| Category |  | Adverse events (%) |  |  |  |  |  |  |  |  |  |  |  |  |  |  |  |
|  | Skin and subcutaneous tissue disorders | Hand-foot skin reaction | 65(41.4%) VS  13(8.3%) | 29 (58%) VS  0% | 71 (46.4% ) VS  10 (6.6%) | 41 (53.2%) VS  0 (0.0%) | NA | 55(43.0%) VS  0% | 37(80%)  VS 0% | 188(82%) VS  16(7%) | 102(50%) VS  0% | 7(29.2%) VS  1(4.0%) | 46(75.4%) VS  0% | NA | 28(66.67%) VS  0% | 12(14.6%) VS  0% | 15(39.5%) VS  0% |
|  | Gastrointestinal disorders | Diarrhea | 87(55.4%) VS  49(31.4%) | 19(38%) VS 0% | 81(52.9%) VS  27 (17.2%) | 11 (14.3%) VS  0 (0.0%) | NA | 76(59.4%) VS  0% | 33(72%)  VS 0% | 71(31% ) VS  11(5%) | 81(41%) VS  0% | 9(37.5%) VS  3(12.0%) | 29 (47.5%) VS  3(1.2%) | NA | 12(28.57%) VS  0% | 5(6.1%) VS  0% | 13(34.2%) VS  4(13.8%) |
|  |  | Nausea | 72 (45.9%) VS  67(42.9%) | NA | 58 (37.9%) VS 59 (39.1%) | NA | NA | 65(50.8%) VS  64(48.5%) | NA | NA | 9(4%) VS  0% | 3(12.5%) VS  2(8.0%) | NA | NA | 2(4.76%) VS  0% | NA | 10(26.3%) VS  3(10.3%) |
|  |  | Vomiting | 23(14.6%) VS  17(10.9%) | NA | 28 (18.3%) VS 40(26.5%) | NA | NA | 65(50.8%) VS  64(48.5%) | NA | NA | 10(5%) VS  0% | NA | NA | NA | NA | NA | 9(23.6%) VS  3(10.3%) |
|  |  | Abdominal pain | 93(59.2%) VS  89(57.1%) | NA | 92(60.1%)  VS 93 (61.6%) | NA | NA | NA | NA | NA | 8(3%) VS  0% | NA | NA | NA | NA | NA | NA |
|  | Investigations | Elevated ALT | NA | NA | 26 (17.0% ) VS  25(16.6%) | 69(89.6%)  VS 55 (77.5%) | NA | 59(49.1%), VS  0% | NA | 48(21%) VS  11(5%) | NA | NA | 20 (32.8%)  VS 45 (18.2%) | NA | NA | NA | NA |
|  | General disorders and administration site conditions | Fever | NA | NA | 59 (38.6%)  VS  52 (34.4%), | 15 (19.5%)  VS  18 (25.4%)， | NA | 41 (32.0%)  VS  42(31.8%) | NA | NA | 21(10%) VS  0% | NA | NA | NA | NA | NA | NA |
|  |  | Fatigue | 127(80.9%) VS  122(78.2%) | 26(52%) VS  0% | 66 (43.1% ) VS 50(33.1%) | 19 (24.7%)  VS  7 (9.9%) | NA | NA | 13(28%) VS  0% | NA | 49(16%) VS  0% | 5 (20.8%) VS  5(20.8%) | 15 (24.6%) VS  47(19.0%) | NA | NA | NA | 10(26.3%) VS  17(58.6%) |
|  | Vascular disorders | Hypertension | NA | 18(36%) VS 0% | 46 (30.1% ) VS 25(16.6%) | 40(51.9%)  VS 28 (39.4%) | NA | 39(30.5%) VS  0% | 6(13%) TACE  0% | 71(31%) VS  16(7%) | 18(9%) VS  0% | NA | 10 (16.4%) VS  2(0.8%) | NA | 8(19.04%) VS  0% | 44(4.9%) VS  0 | 12(31.6%) VS  3(10.3%) |
|  | Respiratory, Thoracic, and Mediastinal Disorders | Voice change | NA | NA | NA | 9 (11.7%)  VS 0 (0.0%) | NA | NA | 1(2%)  VS  0% | NA | 10(5%) VS  0% | NA | NA | NA | 2(4.76%) VS  0% | NA | NA |
|  |  | 1st AU (year) | Takamasa Ohki2015 | Xuying Wan2016  (TACE+sorafenib  VS  TACE) | Wei BAI2013  (TACE+sorafenib  VS  TACE) | Masatoshi Kudo2014  (TACE+Brivanib  VS  TACE+PlaceoA) | Hisashi Hidaka2019  (TACE+orantinib  VS TACE+placebo) | Masatoshi Kudo2017  (TACE+orantinib  VS  TACE+placebo) | Yoshitaka Inaba2013  (TACE+orantinib VS  TACE+placebo) | Zhigang Fu2021  (TACE + Lenvatinib  VS  TACE) | Tao Sun2020  (TACE–apatini  VS  TECA) | Wenzhe Fan2019  (TACE–apatini  VS  TECA) | Juanfang Liu2019  (TACE–apatini  VS  TECA) | Yuanyuan Li2021  (TACE–apatini  VS  TECA) | Zhiyu Qiu2019  (TACE–apatini  VS  TECA) | Xuefeng Kan2020  (TACE–apatini  VS  TECA) | Lujun Shen2020  (TACE–apatini  VS  TECA) |
| Category |  | Adverse events (%) |  |  |  |  |  |  |  |  |  |  |  |  |  |  |  |
|  | Skin and subcutaneous tissue disorders | Hand-foot skin reaction | NA | 8(35.1%) VS  0% | 52(63.4%) VS  0 % | 77 (31%)  VS  5 (2%) | NA | NA | NA | 7 (11.7%) VS  0% | 20 (74.1%)  VS  0% | 45 (52.9%)  VS  0% | 15 (44.1%)  VS  0% | 10 (47.6%)  VS  0% | 20(47.6%) VS  0% | 97 (77%) VS  0% | 27 (67.5%) VS  0% |
|  | Gastrointestinal disorders | Diarrhea | NA | 7(31.0%) VS  0% | 30(36.6%) VS  0 % | 88 (36%)  VS  25(10%) | 78(35.6)  VS  35(16.4) | 123 (28%)  VS  70 (16%) | 20(40 %)  VS  1(2 %) | 11 (18.3%) VS  0% | 10 (37.0%)  VS  0% | 19 (22.1%)  VS  0% | 12 (35.3%)  VS  0% | 6(28.6%)  VS  0% | 11(26.2%) VS  6(7.2%) | 30 (24%) VS  0% | 9 (22.5%) VS  0% |
|  |  | Nausea | NA | 7(2.8%) VS  0% | NA | 70 (28%) VS  69(27%) | 85(38.8)  VS  98(46.0) | 173 (39%)  VS  179 (40%) | NA | NA | NA | NA | 11(32.4%)  VS  13(27.1%) | 9 (42.9%)  VS 7(25.9%) | 9(21.4%) VS  13(15.7%) | NA | NA |
|  |  | Vomiting | NA | NA | NA | 63(26%)  VS  57(23%) | 73(33.3)  VS  65(30.5) | 126(28%)  VS  116(26%) | NA | NA | NA | NA | 11(32.4%)  VS  13(27.1%) | 9(42.9%)  VS 7(25.9%) | 9(21.4%) VS 13(15.7%) | NA | NA |
|  |  | Abdominal pain | NA | NA | NA | 90(37%)  VS  101(49%) | 142(64.%)  VS 130  (61.0%) | 317 (71%)  VS  292 (66%) | 9 (18%)  VS  2(4 %) | NA | NA | 21 (24.7%)  VS  36 (35.0%) | 11 (32.4%)  VS  18(37.5%) | 9(42.9%)  VS 9(33.3%) | 8(19.0%) VS  19(22.9%) | NA | NA |
|  | Investigations | Elevated ALT | NA | NA | NA | 88(36%)  VS  84(33%) | 132(60.3%)  VS  110(51.6%) | 150 (34%)  VS  132 (30%) | 22(44% )  VS  16(31%) | 14 (23.3%) VS  11 (18.3%) | NA | NA | NA | NA | NA | NA | NA |
|  | General disorders and administration site conditions | Fever | NA | NA | NA | NA | 163(74.4%) VS  179(84.0%) | 264(59%)， VS  284(64%) | 24(48%) VS  5(10%) | NA | NA | NA | 10(29.4%)  VS  14(29.2%) | NA | 19(45.2%) VS  51(61.4%) | NA | NA |
|  |  | Fatigue | NA | 5(23.7%) VS  0% | 20 (24.4%) VS  0 % | 101(41%)  VS  59(23%) | NA | 101(23%)  VS  92(21%) | 37(74 % )  VS  10(20%) | 10 (16.7%) VS 0% | 8 (29.6%)  VS  0% | 8 (9.4%)  VS  3 (2.9%) | 12(35.3%)  VS  2(4.2%) | 6(28.6%)  VS  0% | 12(28.6%) VS  21(25.3%) | 28 (22%) VS 0% | 5(20.6%) VS  0% |
|  | Vascular disorders | Hypertension | NA | 8(3.3%) VS  0% | 7 (8.5%) VS  0 % | 116(47%)  VS  29(11%) | 36(16.4%)  VS 36(16.9%) | 47(11%)  VS  39(9%) | 8(16 % )  VS  7(14%) | 29 (48.3%) VS 0% | 14 (51.8%)  VS  0% | 43 (50.6%)  VS 0% | 16 (47.1%)  VS  0% | 11(52.4%)  VS 4(14.8%) | 13(31.0%) VS  2(2.4%) | 58 (46%) VS  0% | 13(32.5%) VS  0% |
|  | Respiratory, Thoracic, and Mediastinal Disorders | Voice change | NA | NA | NA | 45(18%)  VS  5(2%) | NA | NA | NA | 9 (15.0%) VS 0% | 2 (7.4%) VS  0% | 10(11.8%) VS  0% | NA | NA | 4(9.5%) VS  0(0%) | 11 (9%) VS  0% | 5 (12.5%) VS  0% |
| TACE=transarterial chemoembolisation. NA: not available. DEB-TACE:Drug-eluting bead transarterial chemoembolization | | | | | | | | | | | | | | | | | |
